# Design Strategy for a Hydroxide-Triggered pH-Responsive Hydrogel as a Mucoadhesive Barrier to Prevent Metabolism Disorders

**DOI:** 10.1021/acsami.1c17706

**Published:** 2021-12-06

**Authors:** Rui-Chian Tang, Tzu-Chien Chen, Feng-Huei Lin

**Affiliations:** † Department of Biochemical Science and Technology, College of Life Science, National Taiwan University, No. 1, Sec. 4, Roosevelt Rd., Taipei 10617, Taiwan (ROC); ‡ Department of Biomedical Engineering, College of Medicine and College of Engineering, National Taiwan University, No. 49, Fanglan Rd., Taipei 10672, Taiwan (ROC); § Institute of Biomedical Engineering and Nanomedicine, National Health Research Institutes, No. 35, Keyan Rd., Zhunan, Miaoli County 35053, Taiwan (ROC)

**Keywords:** hydrogel, pH-responsive, hydroxide, intestine, metabolism disorders, pectin

## Abstract

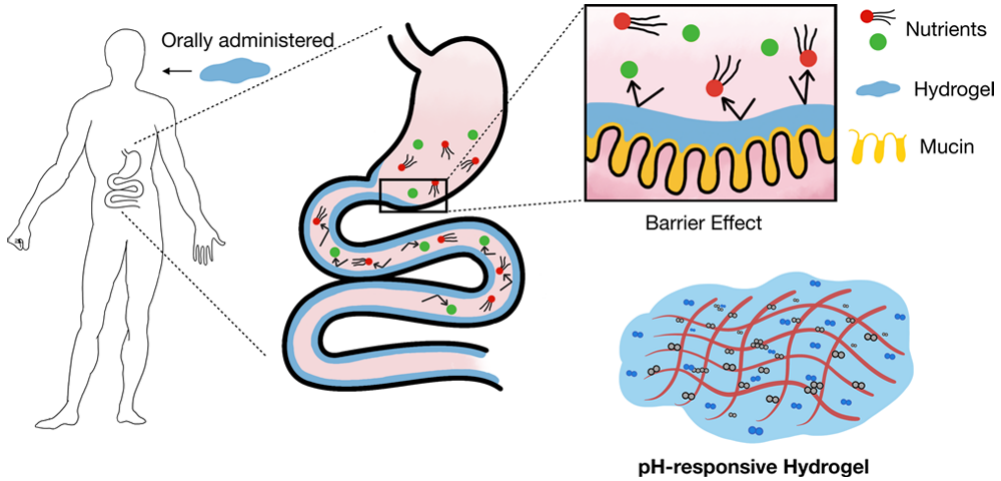

Excess nutrient uptake is one of the main factors of complications related to metabolism disorders. Therefore, efforts have emerged to modulate nutrient transport in the intestine. However, current approaches are mainly invasive interventions with various side effects. Here, a pH-responsive hydrogel is formulated by acidifying the hydroxide compounds within sucralfate to allow electrostatic interactions between pectin and aluminum ions. The pH responsiveness relies on the alternation of cations and hydroxide species, providing reversible shifting from a hydrogel to a complex coacervate system. It acts as a transient physical barrier coating to inhibit intestinal absorption and changes the viscosity and barrier function in different parts of the gastrointestinal tract, showing enhanced mucoadhesive properties. The therapeutic hydrogel remarkably lowers the immediate blood glucose response by modulating nutrient contact with bowel mucosa, suggesting potential in treating diabetes. In addition, it significantly reduces weight gain, fat accumulation, and hepatic lipid deposition in rodent models. This study provides a novel strategy for fabricating pH-responsive hydrogels, which may serve as a competent candidate for metabolism disorder management.

## 1 Introduction

Excess nutrition uptake can lead to obesity, impaired glucose response, and dyslipidemia, which are generally termed under the umbrella of metabolic syndrome.^1^ Numerous comorbidities such as type II diabetes and nonalcoholic fatty liver disease (NAFLD) are closely related.^2^ However, no matter how much the nutrient intake, metabolism disorders depend highly on how much one absorbs. As the chief location for digestion and absorption, the intestine plays a leading role in the nutrient uptake.^3^ Therefore, regulating intestinal absorption could be one of the effective ways to treat metabolism disorders. For example, the Roux-en-Y gastric bypass (RYGB) operation shows more promising results than traditional pharmaceuticals,^4^ providing adequate weight loss and glycemic control. Nevertheless, invasive bariatric surgery irreversibly alters the gastrointestinal tract, deterring more than 98% of the eligible patients.^5^

**Figure 1:**
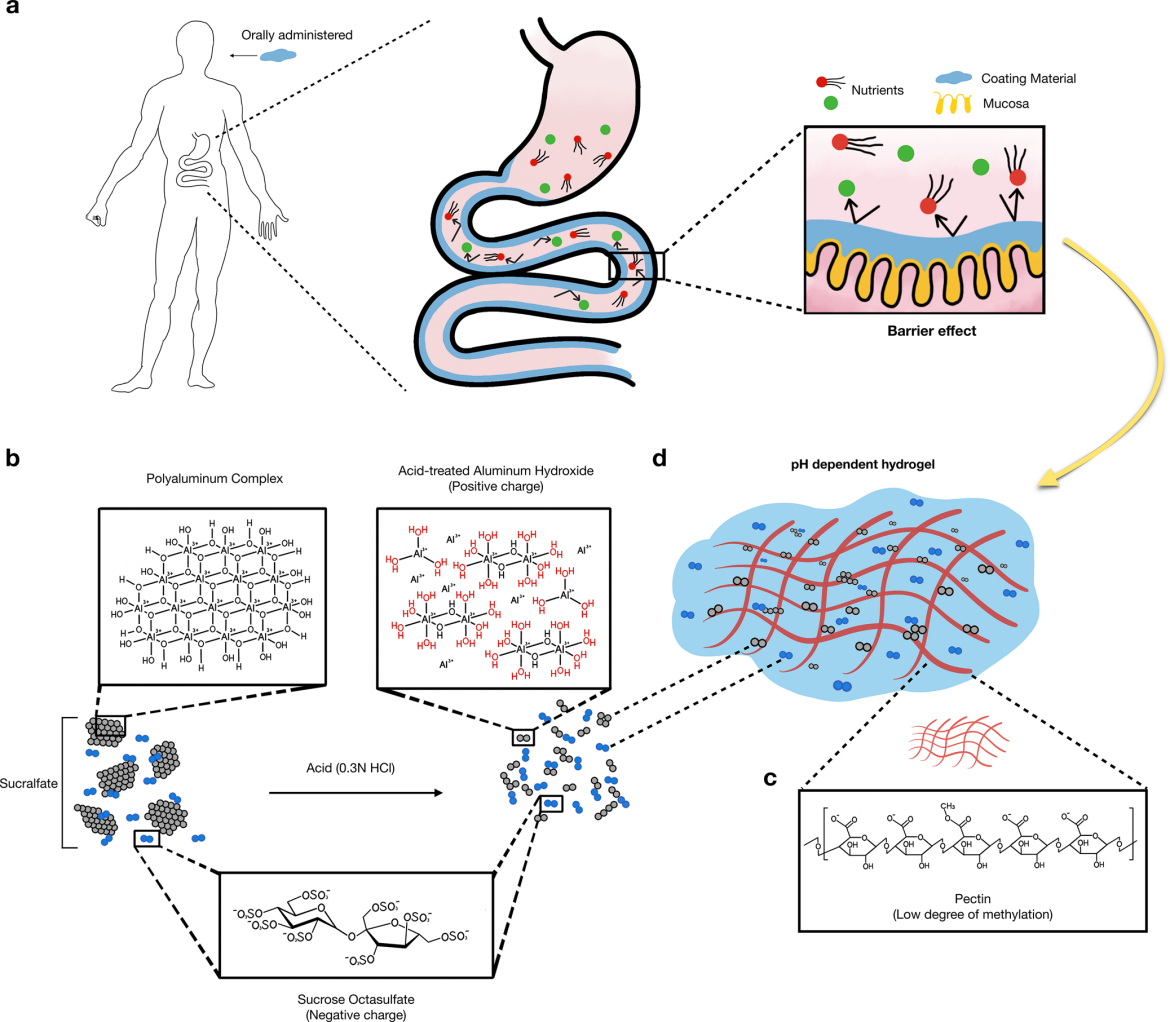
Schematic illustrations of the idea and materials used in this study. (a) Schematic illustrations of an orally administered polyelectrolyte hydrogel as a transient barrier to inhibit excess nutrient uptake. (b) Illustrative schematic of the chemical structure of sucralfate and acid-treated sucralfate. (c) Schematic illustration of the chemical structure of pectin. (d) Schematic representation of the pH-responsive hydrogel composed of pectin and acid-treated sucralfate.

Therefore, a less invasive procedure, EndoBarrier, was first introduced to clinical studies in 2007.^6^ It is an endoscopically implanted device anchored in the upper part of the proximal duodenum. The duodenojejunal polymer sleeve would act as a physical barrier to prevent contact between the ingested food and the intestinal mucosa.^7^ Though helpful in achieving improvements in metabolic parameters, EndoBarrier requires annual removal, and numerous side effects, such as nausea, gastrointestinal bleeding, and even device migration have been reported.^8^ Consequently, a simultaneously safe and effective alternative for bowel inhibition is needed. Thus, we aim to develop an edible and biodegradable supplement that forms a transient covering on the gastrointestinal tract as a temporary barrier to prevent excessive absorption (Figure 1a). The covering mimics the bypassing effect of bariatric surgeries and duodenal sleeves, without requiring patients to undergo these invasive interventions.

In this study, a mucoadhesive material would be considered to serve as a covering on the gastrointestinal tract. Sucralfate is an FDA-approved, orally administered polymer consisting of sucrose octasulfate and polyaluminum complex. It inactivates pepsin, adsorbs bile salts, and exhibits cytoprotective properties to maintain epithelial integrity.^9^ Researchers have also shown that treatment with HCl solution breaks the hydroxyl bonds of the polyaluminum complex into shorter chains (Figure 1b). These positively charged short chains bind to sucrose octasulfate via electrostatic interactions and form a complex coacervate system, which binds to the gastrointestinal tract as a coating layer to reduce blood glucose response through its barrier effect.^10^ However, the daily dosage of sucralfate to achieve glycemic control would be costly. In addition, although relatively rare, constipation, nausea, and urticaria may occur as side effects under standard dosing.^11^ To address this issue, we seek to reduce the amount of sucralfate required while enhancing the barrier properties via creating complex coacervation with charged polymers.

Natural mucoadhesive polysaccharides are of interest for their beneficial effects on the gastrointestinal tract and low cost. Pectin, a component of plant cell walls, was selected because it promotes mucus secretion and maintains epithelial integrity,^12^ prohibiting the infiltration of toxic lipopolysaccharides, which may induce mild inflammation of the intestine and lead to a propensity for obesity and insulin resistance.^13^ Most importantly, it consists of partially methoxy-esterified galacturonic acid units, which makes it an acidic polysaccharide with a p*K*_a_ of about 3.5 (Figure 1c).^14^ Given this property, pectin can form a hydrogel by attracting multivalent cations through its deprotonated carboxyl group at a specific pH, creating a 3D network structure.^15^

Therefore, we predicted that pectin would form a hydrogel with only traces of the acid-treated sucralfate via the attraction between deprotonated carboxyl groups and aluminum ions, forming a complex coacervate system with tight networking barrier structures (Figure 1d). Since adjusting pH values alters the ratio of aluminum cations and precipitated hydroxide species as well as the number of deprotonated carboxyl groups, we hypothesized that this unconventional hydrogel/complex coacervate would possess pH-responsive properties in terms of morphology and barrier function.

In this study, we synthesized the pectin sucralfate hydrogel (PSH) with unique pH-responsive properties and enhanced barrier function. Furthermore, we proposed a pH-responsive gelation mechanism to serve as a design strategy to predict and fabricate various hydrogels with similar behaviors. Finally, we evaluated the therapeutic effects of PSH on ameliorating oral glucose tolerance response, weight gain under a high-fat diet, body fat accumulation, hepatic lipid deposition, and other metabolism disorders.

## 2 Results and Discussion

### 2.1 Fabrication and Physicochemical Properties of PSH

**Figure 2:**
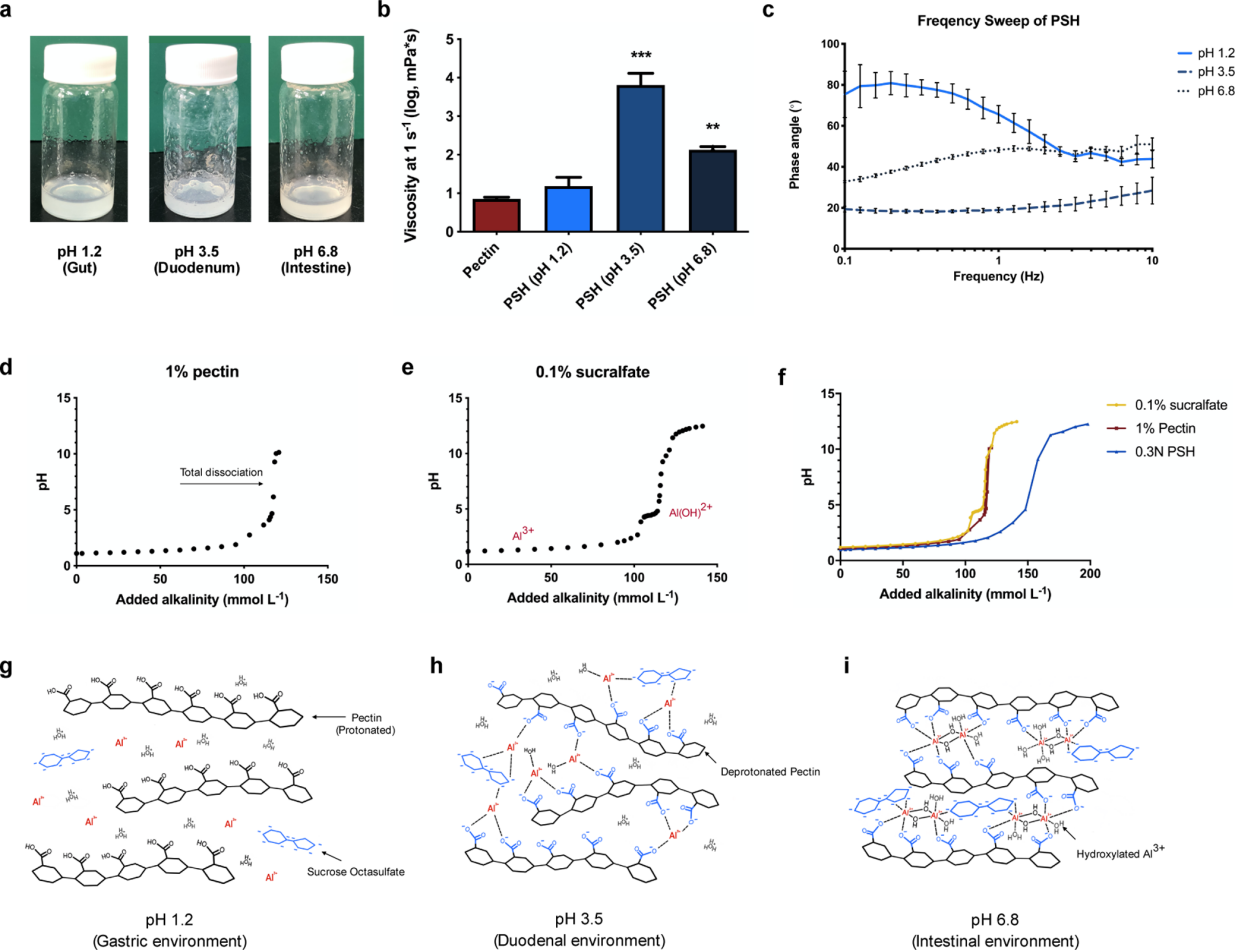
Fabrication, physicochemical properties, and pH-dependent mechanism of PSH. (a) Representative pictures of PSH in different gastrointestinal pH (gut, pH 1.2; duodenum, pH 3.5; intestine, pH 6.8). (b) Viscosity of pectin and PSH in different gastrointestinal pH (gut, pH 1.2; duodenum, pH 3.5; intestine, pH 6.8). One-way ANOVA with multiple comparisons (*n* = 3 per arm, **P < 0.002, ***P < 0.0001 compared to pectin). (c) Change of rheological properties of PSH in different gastrointestinal pH (gut, pH 1.2; duodenum, pH 3.5; intestine, pH 6.8) (*n* = 3). (d) Titration curve of 1% pectin. (e) Titration curve of 0.1% acid-treated sucralfate. (f) Titration curve of 0.1% sucralfate, 1% pectin, and 1% PSH. (g) Proposed scenario for PSH in simulated gut fluid (pH 1.2), where the protonated pectin cannot attract the aluminum ions. (h) Proposed scenario for PSH in simulated duodenal fluid (pH 3.5), where the deprotonated pectin forms a hydrogel with the aluminum ions. (i) Proposed scenario for PSH in simulated intestinal fluid (pH 6.8), where the hydrogel structure decomposes due to the hydroxylation of aluminum ions, leading to the formation of a complex coacervate system.

PSH was synthesized by combining acidified sucralfate in HCl (0.2% w/v) and the same volume of citrus pectin solution (2% w/v) to give a final concentration of 1% PSH (1% pectin plus 0.1% acidified sucralfate). Specifically, pectin with over 74% of galacturonic acid was chosen to provide more carboxyl groups for electrostatic interaction between the polyelectrolytes. PSH was adjusted to pH 1.2, 3.5, and 6.8 to simulate the environments of the stomach, duodenum, and intestine, respectively. In different environments, PSH changed its viscosity, becoming more viscous and gel-like, resembling a mixture of sticky paste and smashed jelly at pH 3.5 (Figure 2a). In contrast, neither the pectin itself nor the pectin added with untreated sucralfate changed (Figure S1, Supporting Information). This suggests that pectin and acid-treated sucralfate can indeed interact to form a hydrogel and that the pH-dependent property is only involved in the formation of PSH. We found that within a pH range of about 0.8–9, the change in viscosity was reversible. However, when the environment became too basic, PSH turned yellow and became much less viscous, and the pH-responsive property was lost (Figure S2, Supporting Information). This could be due to the β-elimination reaction that cleaves the polymeric structure of pectin into smaller fragments that are unable to form a tightly packed hydrogel.^16^

Further rheological analysis showed that PSH viscosity increased slightly at pH 1.2 compared to pectin, became much more viscous at pH 3.5, and reached an intermediate viscosity at pH 6.8 (Figure 2b). This suggests that PSH is harder to be washed away compared to pectin in the duodenum and intestine, where most enzymatic reactions and nutrient absorption occur.^17^ We also found that the concentration of HCl used to treat sucralfate did not affect the viscosity (Figure S3, Supporting Information).

Moreover, the phase angle of PSH at pH 6.8 was higher than that of PSH at pH 3.5 (Figure 2c). The phase angle measures the presence of solid behavior in a viscoelastic fluid in oscillatory rheological analysis. Therefore, the result implies that PSH exhibits more fluidlike rheological properties in a simulated intestinal environment and may form a more conformable coating on the intestinal epithelium. Also, the storage and loss modulii of PSH convey a similar concept (Figure S4, Supporting Information).

### 2.2 pH-Responsive Mechanism of PSH

Typical polysaccharide cation hydrogels, such as alginate hydrogel cross-linked only by calcium ions, do not exhibit such drastic morphological and rheological alternations as PSH at this range of pH value.^18^ The unique pH-responsive property might be attributed to the degree of pectin ionization and the change in available aluminum cation species. For most pectins, their p*K*_a_ values are approximately 3.5,^14^ and over 99% of carboxyl groups would be deprotonated at pH > 4.5 (Figure 2d). Furthermore, the p*K*_a_ value of the first hydroxylation for aluminum ions is 5.02.^19^ Thus, for acidified sucralfate, aluminum ions are abundant at lower pH; at pH 4–5, most of them convert into aluminum hydroxide species (mainly Al(OH)^2+^),^20^ corresponding to the lower horizontal part of the titration curve (Figure 2e).

We combine the titration curves and propose each scenario at different pH values (Figure 2f). In the gastric environment, despite the presence of aluminum ions, the pectin does not have sufficient deprotonation of the carboxyl group to develop into a hydrogel (Figure 2g). In the duodenal environment, the negative ionized carboxyl groups bind to the positive aluminum ions to convert into a hydrogel (Figure 2h). The viscosity of PSH peaks at pH 4–5 and gradually decreases as the aluminum ions are hydroxylated into aluminum monohydroxide, which may be structurally too large to be retained in the hydrogel structure. Finally, the oppositely charged polyelectrolytes transform into a complex coacervate system in the intestinal environment (Figure 2i).

The observation of the titration curve could serve as a template to predict new pH-responsive hydrogels consisting of anionic gelling polymers and materials with multivalent cationic hydroxide. Gelation occurs when the number of cations and deprotonated anionic groups is sufficient, while the hydrogel structure decomposes once the cations begin to precipitate at a pH value equal to the first hydroxylation of cations. That is, the formed hydrogels would transform into a complex coacervate system.

Various existing hydroxides, whether with divalent or trivalent ions, may serve as possible candidates. Furthermore, manipulating the ratio of gelling polymers and hydroxides changes the amount of alkali (or acid) needed to trigger hydrogel transformation. This suggests the possibility of fine-tuning the reversible pH-responsive property in a targeted environment.

### 2.3 Barrier Function

**Figure 3:**
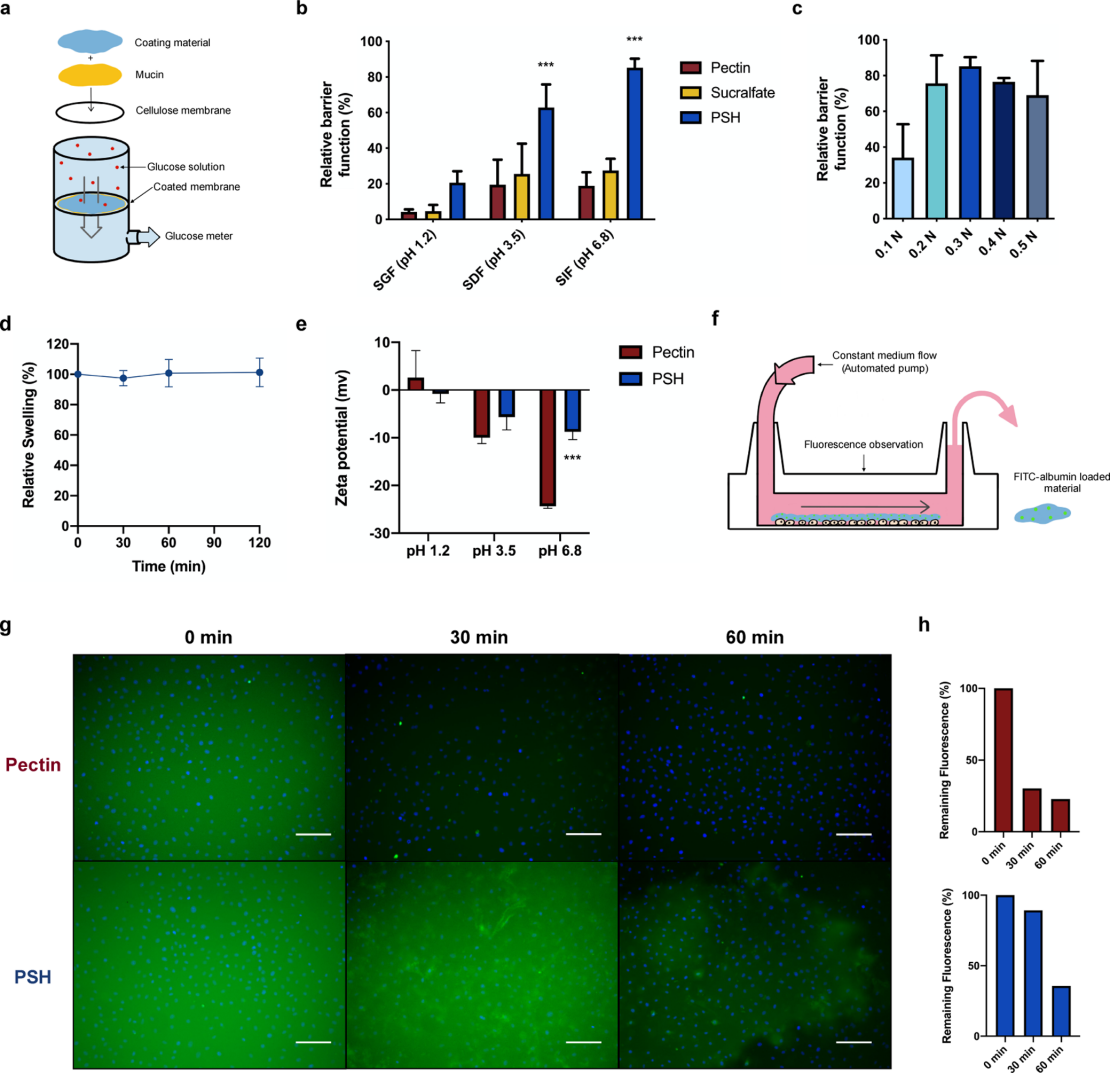
Barrier function, swelling, and adhesion assessments of PSH in vitro. (a) Schematic representation of the barrier function test. 1% of the material was applied on a mucin-coated cellulose membrane and mounted onto a microfiltration laboratory apparatus, and the glucose permeation was measured using a glucose meter. (b) Relative barrier function of pectin, acid-digested sucralfate, and PSH tested with glucose solution (500 mg dL^–1^) in different gastrointestinal pH (gut, pH 1.2; duodenum, pH 3.5; intestine, pH 6.8). Two-way ANOVA with multiple comparisons (*n* = 3 per arm, ***P < 0.0001 compared to pectin). (c) Relative barrier function of PSH formed with sucralfate digested in HCl solution (0.1–0.5 N) tested with glucose solution (500 mg dL^–1^) in intestinal pH (*n* = 3). (d) Swelling of PSH in simulated duodenal fluid over time (*n* = 3). PSH was applied evenly on a mucin-coated membrane and soaked in simulated duodenal fluid at 37 °C. Relative wet weights were measured and plotted with time as the *x*-axis. (e) Zeta potential of pectin and PSH in different gastrointestinal pH (gut, pH 1.2; duodenum, pH 3.5; intestine, pH 6.8). Two-way ANOVA with multiple comparisons (*n* = 3 per arm, ***P < 0.0001). (f) Schematic illustration of the in vitro adherence evaluation. FITC-albumin encapsulated materials were loaded on a μ-slide seeded with IEC-6 cells and subjected to a constant medium flow. The retained fluorescence was observed using a fluorescence microscope. (g) Mucus adhesion of pectin and PSH after being washed by medium for periods of time. FITC-albumin encapsulated materials are represented in green; the nucleus is represented in blue. Scale bar, 100 μm. (h) Remaining fluorescence over time. Fluorescence at each time point was normalized to the initial fluorescence of pectin and PSH (100%).

Improvements in the barrier properties of PSH were investigated. The materials used in the study spread on a mucin-coated porous cellulose membrane were subjected to gravity loading, followed by a permeability test with a d-glucose solution (Figure 3a). Glucose was chosen as the main target for the effect on type II diabetes. As shown in Figure 3b, acidified sucralfate (33% blocked) exhibits a slightly better barrier effect than pectin (26% blocked). Interestingly, the permeability changes notably at different pH values for PSH. Although PSH exhibits lower barrier properties in the simulated gut environment (21% blocked), it shows remarkably enhanced barrier function in simulated duodenal and intestinal environments (up to 63 and 85%, respectively). The result serves as a key implication that PSH has the best barrier effect in environments where digestion and nutrient absorption occur the most, which is highly favorable in this study.

Intriguingly, the highest viscosity of PSH at pH 3.5 does not provide the highest barrier function. Instead, PSH at pH 6.8 inhibits glucose permeation the most. From the proposed mechanism, we deduced that it is because when pectin nearly reaches total ionization, the attraction between cationic aluminum hydroxide species and sucrose octasulfate along intermolecular hydrogen bonds peaks to form a highly packed complex coacervate system.

The influence of the acid concentration used to treat sucralfate on the barrier function was investigated. For digestion of sucralfate, 0.1–0.5 N HCl was used, and the corresponding PSH was adjusted to pH 6.8 since our interest lay in the formation of an intestinal barrier. The relative barrier function was the best at 0.3 N (Figure 3c); therefore, sucralfate was treated with this concentration to form PSH in subsequent experiments.

### 2.4 Swelling and Zeta Potential

Given that PSH becomes hydrogel-like at duodenal pH (pH 3.5), we were curious if PSH would swell under the condition. Therefore, PSH was evenly applied to a mucin-coated membrane and soaked in simulated duodenal fluid at 37 °C. The hydrogel layer on the mucus surface hardly swelled for at least 2 h in simulated duodenal fluid (Figure 3d). This indicates that the PSH layer coated on the intestinal tract retains its original shape and the barrier properties change minimally during meals.

Since the shift in electric charge plays an important role in the pH-responsive property, we investigated the change in zeta potential of pectin and PSH at different pH values. With increasing pH, the zeta potential of pectin dropped from +3 mV at pH 1.2 to around −24 mV at pH 6.8, whereas the zeta potential for PSH ranged only from −1 to −9 mV, which was due to the neutralization of the aluminum cation species. When the pH is gradually lowered, the cation species present (Al^3+^, Al(OH)^2+^) is constantly attracted to the increasingly deprotonated carboxyl group; therefore, the zeta potential of PSH was closer to 0 mV compared to pectin (Figure 3e).

Considering that oligosaccharide chains confer a negative charge to mucins through carboxyl and sulfate groups,^21,22^ many studies have shown that polymers or proteins with a lower negative charge are better able to adhere to or penetrate the mucus.^21−24^ Therefore, we reasoned that this lower negativity might be another factor, in addition to higher viscosity, that contributes to PSH adhering more firmly to the luminal surface of the gastrointestinal tract.

### 2.5 Adhesion Assessment of PSH In Vitro

We hypothesized that polyelectrolytes within the coacervate system might bind more strongly to the negatively charged mucin oligosaccharides, further increasing the adhesion of PSH to the intestinal epithelium. Therefore, fluorescence-modified materials were loaded onto a μ-slide seeded with IEC-6 cells, and constant flow-induced shear was imposed on the system to simulate stress under peristalsis (Figure 3f). As shown in Figure 3g, the pectin and PSH showed similar fluorescence intensity before constant shear (0 min). After 30 min, the fluorescence of pectin decreased significantly, and 60 min later, the fluorescence was barely visible. On the other hand, under the same circumstances, a much larger proportion of PSH remained on the μ-slide, indicating that PSH has better adhesion to mucus than pectin.

### 2.6 Evaluation of Mucus Adherence In Vivo

**Figure 4:**
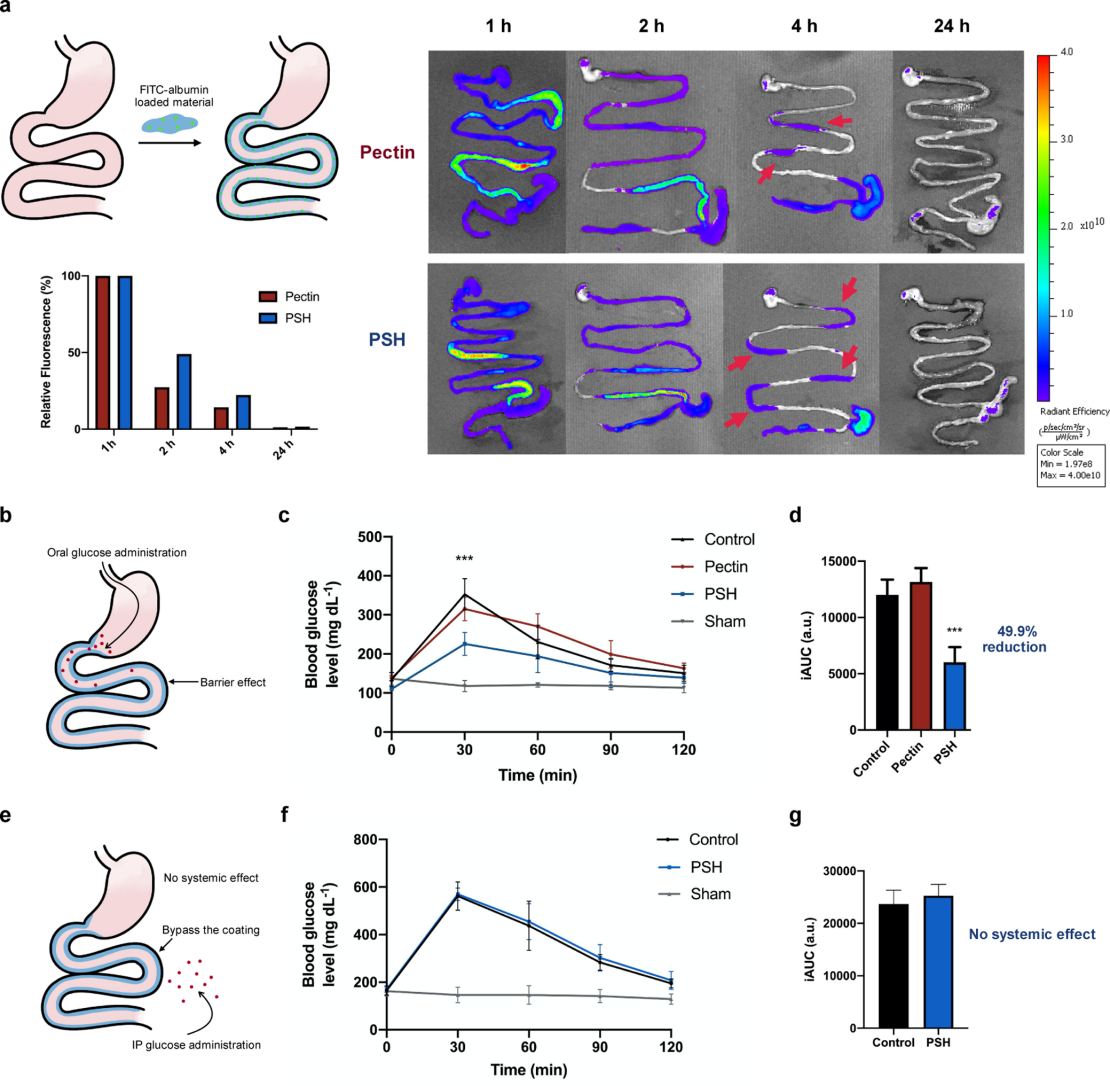
Evaluation of mucus adherence in vivo and the reduced glucose response with PSH administration in mice. (a) Schematic illustration of the in vivo mucus adhesion evaluation and the fluorescence image analysis. Mice were gavaged with materials (250 mg per kg mouse) encapsulated with FITC-albumin, and the gastrointestinal tracts from the stomach to the colon were harvested in 1, 2, 4, and 24 h and imaged using an IVIS in vivo imaging system. Mice gavaged with PBS were used as the control. (b) Schematic representation of the barrier effect of PSH in OGTT with oral glucose administration. (c) OGTT curves of mice gavaged with pectin and PSH (250 mg per kg mouse). Mice gavaged with PBS were used as the control; mice without glucose administration were used as shams. Two-way ANOVA with multiple comparisons (*n* = 4, ***P < 0.0001). (d) iAUC of the OGTT curves. One-way ANOVA with multiple comparisons (*n* = 4 per arm, ***P < 0.0001 compared to control). (e) Schematic representation of the no systemic effect of PSH in IPGTT where IP glucose administration bypassed the barrier coating. (f) IPGTT curves of mice gavaged with PSH (250 mg per kg mouse). Mice gavaged with PBS were used as the control; mice without glucose administration were used as shams. Two-way ANOVA with multiple comparisons (*n* = 4, ***P < 0.0001). (g) iAUC of the IPGTT curves. One-way ANOVA with multiple comparisons (*n* = 4 per arm).

C57BL/6 mice were gavaged with albumin FITC-loaded candidate materials to test their ability to resist intestinal peristalsis over an extended period in vivo. The intestines were harvested at different time points and visualized using the IVIS system (Figure 4a). Fluorescent signals were detected mainly in the duodenum and intestine within 1 h for both PSH and pectin. The signal intensities gradually faded out with time, and a key point to notice was at the fourth hour. At this time point, much more PSH remained in the upper part of the intestine compared to the small amount of pectin fluorescence retained. The observation was also confirmed by quantifying the relative fluorescence intensity. Thus, we conclude that PSH indeed binds more strongly to the intestinal mucus than pectin itself, in agreement with the results of the in vitro μ-slide test. Moreover, there was no fluorescence signal after 24 h, which indicated that PSH would only form a transient barrier on the gastrointestinal tract, without continuing to accumulate in the body.

### 2.7 Reduced Glucose Response with PSH Administration in Mice

We then evaluated the in vivo barrier effect of PSH on nutrients, particularly glucose, for our interest in metabolic syndrome-related diseases. An oral glucose tolerance test (OGTT) was performed to determine whether the PSH on the gastrointestinal mucosa could act as a physical barrier to lower the blood glucose response (Figure 4b). The initial blood glucose levels of both pectin and PSH were similar to the control group, indicating that the materials were not digested into absorbable fragments to influence blood glucose. As shown in Figure 4c,d, PSH showed a remarkable reduction in blood glucose response, with a 49.9% reduction in iAUC compared to the saline control, which supports its feasibility as a nutrient barrier. Moreover, pectin did not show the same ability to inhibit glucose absorption, indicating the superior performance of the engineered PSH.

Concerning metabolism disorders, dramatic fluctuations in blood glucose are associated with various problems, including coronary heart disease and aorta endothelial cell apoptosis.^25,26^ As opposed to the steep rise and fall in the control group, where the highest blood glucose level was at around 350 mg dL^–1^ and quickly dropped to 230 mg dL^–1^, the curve of the PSH-fed group rose steadily to merely 230 mg dL^–1^ and fell more slowly. This demonstrated the ability of PSH to keep a steady blood glucose level after food intake, which is especially vital for patients with diabetes.

An intraperitoneal glucose tolerance test (IPGTT) was performed to examine whether PSH has a systemic effect on blood glucose since glucose administration bypasses the physical barrier of the PSH in the gastrointestinal tract (Figure 4e). In the IPGTT, the blood glucose level of both the control and PSH groups rose to around 560 mg dL^–1^, and there was no difference in the glucose response curves (Figure 4f), which indicates that the reduction in glucose response was due to the localized barrier effect rather than a systemic effect.

The mucoadhesion of materials mostly depends on the interfacial mucosa-material relationship. Given the electric buffering properties that mucin possess, it will potentially affect the physicochemical properties of materials, especially charged colloidal systems such as PSH. Taking the complex and dynamic environment into consideration, foods with various pH values may also influence the structure of PSH. Moreover, the interplay between mucus, PSH, and nutrients with different forms (whether liquid, emulsion, or semisolid) can impact the intestinal uptake.^27^ Therefore, more investigation into the microscopic environment of PSH in actual gastrointestinal tracts may allow future optimization of its function in vivo.

### 2.8 Long-Term Therapeutic Effects of PSH

**Figure 5:**
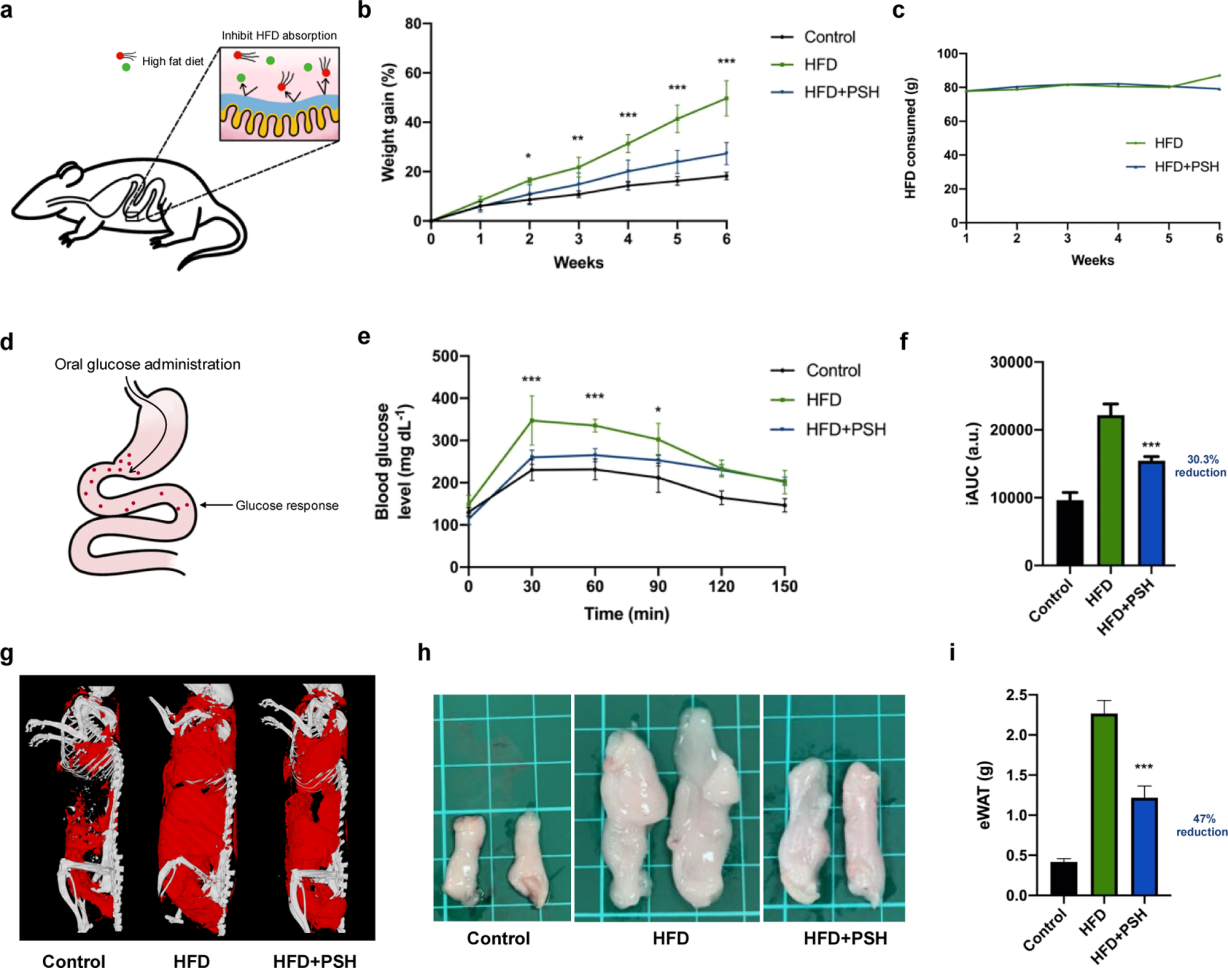
Long-term therapeutic effects of PSH on reducing weight gain, glucose response, and body fat deposition in a HFD-induced mice model. (a) Illustrated schematic of the gastrointestinal barrier of PSH to inhibit HFD absorption in the mice model. (b) Weight gain (%) of mice fed with HFD and HFD + PSH. Mice with rodent chow (regular food) were used as the control. Two-way ANOVA with multiple comparisons (*n* = 6, ***P < 0.0001 for HFD + PSH vs HFD). (c) Food intake of mice fed with HFD and HFD + PSH. (d) Schematic illustration of a standard OGTT to evaluate blood glucose response in mice. (e) OGTT curves of mice administered with glucose. Two-way ANOVA with multiple comparisons (*n* = 6, *P < 0.05, ***P < 0.0001 for HFD + PSH vs HFD). (f) iAUC of the OGTT curves. One-way ANOVA with multiple comparisons (*n* = 6 per arm, ***P < 0.0001 compared to HFD). (g) 3D CT imaging of mice with a resolution of 35 μm voxel spacing. Skeletons are represented in white; adipose tissue iss represented in red. (h) Representative pictures of eWAT. Scale bar, 1 cm. (i) Dry weight of the harvested eWAT. One-way ANOVA with multiple comparisons (*n* = 6 per arm, ***P < 0.0001 compared to HFD).

C57BL/6 mice with 4–8 weeks of age were fed high-fat diet (HFD) to establish an obesity model. PSH was administered orally (125 mg per kg) daily for 6 weeks to investigate its effects on metabolic parameters compared with the HFD and control groups (Figure 5a). As seen in Figure 5b, weight gain was significantly lower in the HFD + PSH group compared to the HFD group from the second week onwards, and the difference became greater as the experiment progressed, eventually leading to a 28.4% decrease in weight gain. Compared to the control group (normal diet), the HFD + PSH group had an increased weight gain by only 9.1% even when given a HFD. In terms of food intake, the HFD + PSH group was almost identical to the HFD group (Figure 5c), suggesting that the reduction in weight gain of the HFD + PSH group was not due to an increased feeling of satiety for mice.

HFD is known to induce insulin resistance in mouse models.^28,29^ Therefore, a standard OGTT (without PSH gavage) was performed to evaluate the blood glucose response after daily administration of the materials for 6 weeks (Figure 5d). In the control and HFD + PSH groups, the initial blood glucose level was merely 130 and 115 mg dL^–1^, respectively, while in the HFD group, it was 150 mg dL^–1^ (Figure 5e). This implies that PSH could improve the diet-induced high fasting blood glucose level. Moreover, the PSH-treated mice showed a notable improvement in the blood glucose response. The peak blood glucose level was only 260 mg dL^–1^ for the HFD + PSH group and near 230 mg dL^–1^ for the control group, and the curve patterns resemble. In contrast to the HFD group, which had a 350 mg dL^–1^ peak value, the HFD + PSH group had a 30.3% reduction in iAUC (Figure 5f). The result suggests that PSH can improve insulin sensitivity in the long term. We deduce that PSH improves insulin sensitivity for several reasons. First, PSH was synthesized mainly by pectin, which promotes mucus secretion and maintains epithelial integrity. Therefore, PSH may inhibit HFD-induced infiltration of toxic lipopolysaccharides that contributes to susceptibility to insulin resistance.^13^ Second, sucralfate present in PSH may also exert cytoprotective properties to enhance epithelial repair by stimulating prostaglandin synthesis.^9^

We further investigated fat accumulation to determine the effect of HFD + PSH on body composition under HFD. A 3D computed tomography (CT) imaging system was used to visualize the total amount of adipose tissue in mice. As shown in red, for the adipose tissue, HFD + PSH reduced its deposition in the hip, abdominal, chest, and back regions of the mice (Figures 5g and S11). The decreased visceral fat formation was also confirmed by measuring the epididymal white adipose tissue (eWAT) weight. The eWAT of PSH-fed mice was only 53% the weight of the HFD group (Figure 5i). These results suggest that PSH reduces the formation of total and visceral fat. However, the total amount of adipose tissue and the eWAT of the control group were significantly less when compared to HFD + PSH. The result may indicate that although PSH showed a promising effect in reducing weight gain and fasting blood glucose level under a HFD, reversing body fat accumulation to match the normal diet group might be challenging.

Through histological analysis, hypertrophy of white adipocytes was observed in the HFD group, while adipocyte sizes were relatively smaller in the HFD + PSH group, in agreement with the macroscopic observation mentioned above (Figure S12).

### 2.9 Effects of PSH on NAFLD

**Figure 6:**
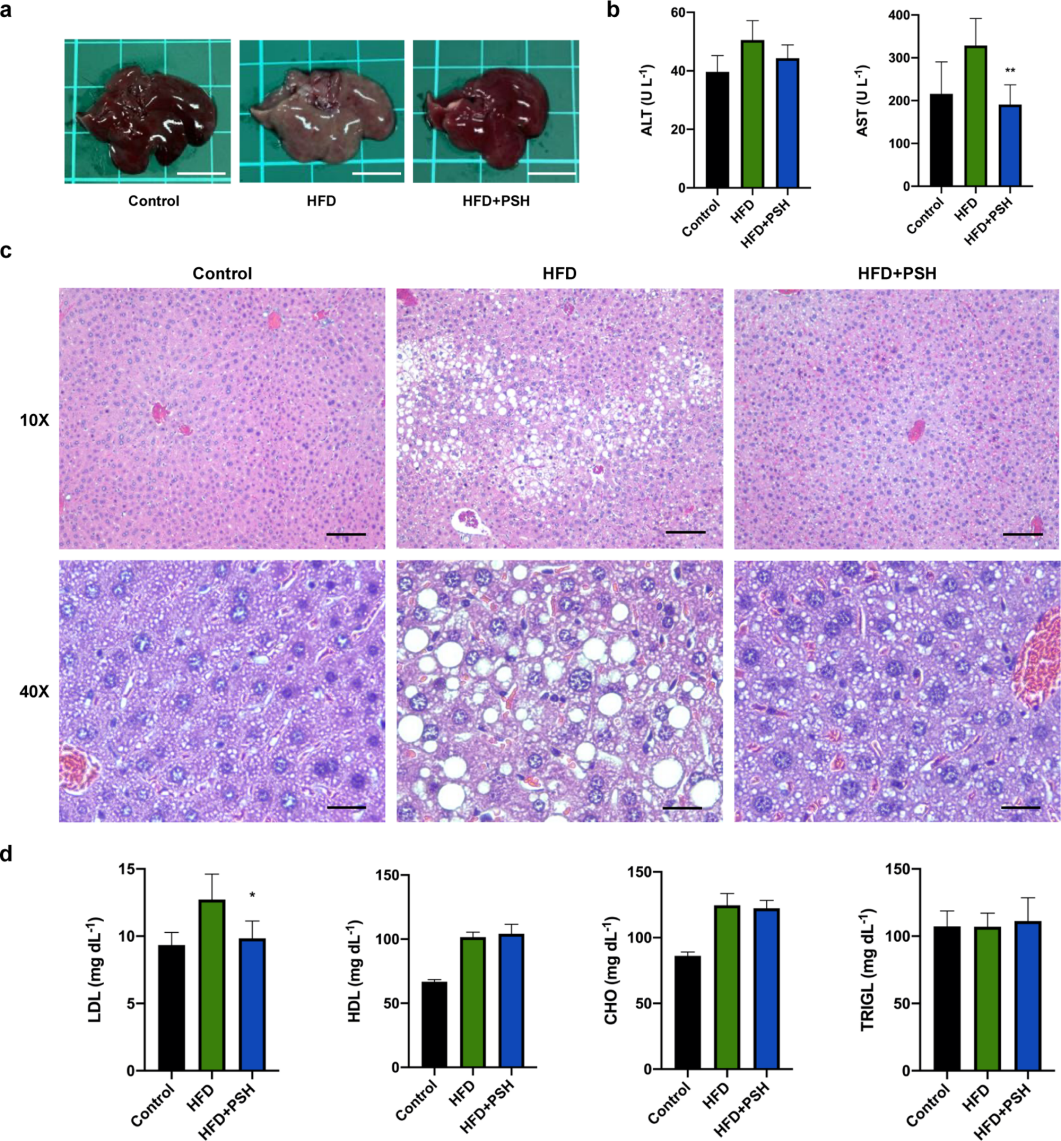
Therapeutic effects of PSH on attenuating fatty liver and LDL metabolism. (a) Representative pictures of livers. Scale bar, 1 cm. (b) Biochemical markers for liver injury assessments. One-way ANOVA with multiple comparisons (*n* = 6 per arm, **P < 0.002 compared to HFD). (c) Histological analysis of the liver section. Scale bar, 100 μm for 10× view; 25 μm for 40× view. (d) Biochemical markers for lipid metabolism. One-way ANOVA with multiple comparisons (*n* = 6 per arm, *P < 0.05 compared to HFD).

NAFLD, a feature of metabolic syndrome,^30^ is characterized by the accumulation of triglycerides and cholesteryl esters in hepatocytes. It is due to a constant imbalance between fatty acid influx and triglyceride utilization.^31^ Given the barrier properties of PSH, we speculated that it might inhibit excess fat uptake and thereby reduce lipid deposition in the liver. Morphologically, the liver of the HFD group was pale in color, while the HFD + PSH group had a reddish shade similar to the control group (Figure 6a). Serum biochemical analysis showed that the HFD group developed higher aspartate aminotransferase and alanine aminotransferase, which are widely used as enzyme biomarkers of liver injury.^32^ In contrast, the HFD + PSH group had a profile similar to that of the control, suggesting that PSH may attenuate liver injury correlated with excessive triglyceride aggregation (Figure 6b).^33^ The histological analysis allows direct observation of hepatic fat accumulation. The HFD group had more and enlarged lipid droplets (white bubbles), while the HFD + PSH group had smaller lipid droplets. The result shows that PSH remarkably decreases hepatic fat deposition despite HFD (Figure 6c).

Elevated plasma low-density lipoprotein (LDL) was found in patients with NAFLD and insulin resistance.^34^ Moreover, LDL is known to lead to cardiovascular diseases.^35^ Therefore, we investigated the ability of PSH to lower serum LDL. As shown, the HFD + PSH group had a remarkable decrease in atherogenic LDL compared with the HFD group, although the difference in HDL and other related biochemical markers was not significant (Figure 6d). Therefore, we deduced that PSH might reduce lipid accumulation in the liver and further improve fat metabolism.

We showed that PSH forms a transient coating on the gastrointestinal tract and essentially mimics the crucial part of proximal bowel isolation of RYGB and EndoBarrier in a noninvasive way. However, a majority of current gastric bypass research is based on established obesity or type II diabetes models.^36,37^ That is, the treatment comes after the disease. On the contrary, our current research aims to prevent the formation of metabolic complications in an earlier state. Therefore, future studies may consider starting PSH treatment after establishing obesity or diabetes models to give a more comprehensive understanding of the efficacy of PSH compared to existing solutions.

Concerning possible aluminum toxicity, no plaque was observed in the histological analysis of brains in mice given with PSH for 6 weeks (Figure S15, Supporting Information), indicating that the minimal amount of aluminum ions present would not lead to amyloid-β plaque accumulations in the brain. Furthermore, there is no significant difference in the histological analysis between HFD and HFD + PSH groups. Hence, we concluded that the risk of aluminum toxicity is little to none.

## 3 Conclusions

In summary, acidifying the aluminum hydroxide component within sucralfate attracts deprotonated pectin to form a pH-responsive hydrogel. The viscosity changes as the pH in the gastrointestinal tract varies. By observing the titration curve of the hydrogel, we proposed a possible mechanism for its unique pH-responsive property. Both the barrier function and the adhesion property of the hydrogel were remarkably better when tested in vitro and in vivo as it formed a tightly packed complex coacervate system. When administered daily, PSH reduced weight gain, blood glucose response, fat accumulation, and hepatic lipid storage in the HFD mouse model. Therefore, we concluded that the hydrogel may serve as a competent candidate for the treatment of metabolic syndrome-related complications, such as type II diabetes, obesity, and NAFLD. Due to its ease of use and low cost, this orally administered polymer may appeal to a wider range of patients, even to those with mild disease. Furthermore, the proposed mechanism may give light to the synthesis of various pH-responsive hydrogels containing gelling anionic polymers and multivalent hydroxides.

## 4 Methods

### 4.1 Fabrication of PSH

To convert sucralfate into a complex coacervate system capable of interacting with pectin, sucralfate was treated with 0.3 N HCl (0.2% w/v) for 1 h. Subsequently, 2% w/v of citrus pectin (>74% galacturonic acid, >6.7% methoxy groups, *M*_w_ = 195,000, Sigma-Aldrich) in distilled deionized water (DDW, pH 7) was thoroughly mixed with the same volume of the treated sucralfate for 1 min to obtain the PSH (with a final concentration of 1% pectin and 0.1% treated sucralfate). The PSH was further adjusted to pH 1.2 to maintain its potency and stored at room temperature for use within 1 week.

### 4.2 Rheological Analysis

Rheological properties were measured using a rheometer (TA Instruments, AR2000). A 40 mm plate was chosen to measure the dynamic viscosity of PSH at different pH values (shear rate, 0.1–10 s^–1^ in the logarithmic scale; a shear rate of 1 s^–1^ was chosen to compare the viscosity). The dynamic frequency sweep was used to measure the dynamic phase angle (frequency range, 0.1–10 Hz in logarithmic scale). All measurements were performed at 37 °C to simulate the physiological environment.

### 4.3 Investigation of Barrier Properties Using Mucin-Coated Membrane

Since the mucoadhesive property of the material mainly depends on the interfacial interaction between the mucin and the material, a mucin layer was coated on a porous membrane to mimic the unstirred mucus layer. In addition, commercially available mucin with a fixed concentration was used to obtain reproducible results, given the inhomogeneity of mucin from different sources. A cellulose membrane (pore size 6 μm, Advantec) was incubated in 3% w/v porcine stomach mucin (Sigma-Aldrich) in DDW and gently shaken for 1 h at room temperature. The excess mucin solution was removed with 1 mL of DDW (pH 7). The mucin-coated membrane was used within 1 h. Then, 1 mL of a 1% w/v candidate polymer (10 mg of material) formulation in DDW (pH 7) was drawn with a pipette and applied evenly to cover the whole mucin-coated membrane without leaving any spare space to avoid leakage of glucose solution. The membrane was further tilted to the vertical position for 1 min to eliminate excess material that was not fully adhered to the membrane. An exception to this procedure, sucralfate was dissolved in a 0.3 N HCl. The polymer-coated membrane was mounted onto a microfiltration laboratory apparatus (Spectrum Chemical Mfg. Corp), 50 mL of glucose solution (500 mg dL^–1^) was added, and samples were collected from the container of the apparatus after 10 min. Barrier property tests were performed with three repeats for each material individually. Glucose concentration was measured using a glucometer (Accu-Chek Instant, Roche). Results were normalized to a mucin-coated membrane without the application of a candidate polymer (0% blocked).

### 4.4 Zeta Potential

To evaluate the change in electrical charges for PSH at different pH values, the zeta potential was determined using a 90 Plus/BI-MAS particle size analyzer (Brookhaven Instruments). Measurements were performed under an electric field of approximately 9 and 3 V cm^–1^ for pectin and PSH, respectively. Samples were adjusted to 1% in DDW and heated to 37 °C before analysis. Each sample was measured in triplicate runs in a polystyrene cuvette (replicate analysis), each run consisting of 40 cycles.

### 4.5 In Vitro Adherence Evaluation

The μ-slide pumping system was used to investigate the enhanced adhesion property of PSH with mucin. 70,000 IEC-6 cells were seeded into the μ-Slide I 0.4 Luer (ibidi) and incubated for 24 h to generate sufficient mucin. Then, the cells were stained with Hoechst for 30 min to visualize the nucleus and washed three times with 100 μL of DMEM to remove excess stain. Then, 100 μL of the 1% candidate material (adjusted to pH 7) encapsulated with FITC-albumin (10% of material dry weight) was applied and incubated with mucin for 30 min (equivalent to 1 mg of candidate material containing 0.1 mg of FITC-albumin). The μ-slide was mounted on a programmable syringe pump (New Era Pump Systems) to stimulate shear force during intestinal peristalsis. Cells were under a constant flow of DMEM at a rate of 150 μL min^–1^, which produced a shear stress of 1.8 μN cm^–2^. The materials were observed with a fluorescence microscope at the time points of 0, 30, and 60 min. The remaining fluorescence over time was quantified using ImageJ and normalized to the initial fluorescence of pectin and PSH (100%).

### 4.6 Fluorescence Imaging of the Gastrointestinal Tract with PSH Gavage

To investigate the increased adherence of PSH in the gastrointestinal tract, C57BL/6 mice were gavaged with PSH or with pectin (dose, 250 mg per kg mouse) encapsulated with FITC-albumin (10% of effective dry weight), and the gastrointestinal tracts were harvested after 1, 2, 4, and 24 h. Gastrointestinal tracts were then imaged using an IVIS Lumina II in vivo imaging system (PerkinElmer). Mice with phosphate-buffered saline (PBS) administration were used as controls, and all images were normalized using the control.

### 4.7 OGTT and IPGTT

To evaluate the in vivo effect of PSH on postprandial glucose uptake, C57BL/6 mice at 4–6 weeks of age were administered PSH, followed by an OGTT. Based on the recommendation of IACUC, the maximum gavage volume is 10 mL per kg mouse. Given an average weight of 25 g for a mouse, the maximum volume is 250 μL. Furthermore, PSH became too viscous to be administered through the gavage needle when exceeding 2.5%. Therefore, the dosage was 250 μL of 2.5% PSH for each mouse, which was 250 mg per kg mouse (i.e., 6.25 mg dose per mouse), and the PSH was adjusted to around pH 7. According to the human equivalent dose calculation based on the body surface area recommended by FDA, the human equivalent dosage of 250 mg per kg mice is a 20.3 mg per kg human dose. If we consider a 60 kg human, the PSH dosage is ∼1.2 g containing ∼1.1 g of pectin and 0.1 g of sucralfate. The dosage is much less than the clinical pectin usage of 10–20 g daily and the maximum sucralfate dosing of 4–5 g daily. The dosage may be further reduced through optimization. In the OGTT experiments, C57BL/6 mice were fasted overnight (starting at 4 pm with access to water, fasting duration 18 h before treatment) and gavaged with PSH, pectin, or PBS. Then, 1 h after material administration, a 0.5 g per mL glucose solution was administered at a dose of 3 g per kg mouse to measure the changes in glucose levels for 120 min (*n* = 4). Mice without glucose administration were used as shams. Blood was collected from the tail vein to measure glucose levels using a glucometer (Accu-Chek Instant, Roche). Each data point was plotted with time as the *x*-axis, and iAUC was calculated based on the plot and preadministration glucose level as the baseline. Statistical difference was determined using two-way analysis of variance (ANOVA). Results were considered significant when *P* ≤ 0.05 (***P < 0.0001). In IPGTT, mice were treated the same as in the OGTT, except that the glucose was injected directly into the peritoneum.

### 4.8 Investigation of the Therapeutic Effects of PSH Long Term

C57BL/6 mice at 4–8 weeks of age were fed a HFD consisting of 60% kcal fat (Dyets) to establish the obesity model. Mice with rodent chow only were used as the control group. Pectin and PSH were administered via oral gavage at a dose of 125 mg per kg mouse daily for 6 weeks, and the PSH was adjusted to around pH 7. DDW was gavaged daily to the control and HFD groups to provide the same stress conditions. Body weight and food intake were recorded once a week. Mean values for each group were plotted with time as the x-axis. In OGTT conducted after 6 weeks of daily gavaging, mice were treated the same as previously mentioned, except that PSH was not given before glucose administration.

### 4.9 Animals

All animal testing protocols were approved by the National Taiwan University Institutional Animal Care and Use Committee (20201042). Male C57BL/6 mice (LASCO) of similar age were housed in groups (five per cage) on a 12 h light–dark cycle; rodent chow and water were given ad libitum. Mice were acclimatized for 1 week before performing the following experiments.

## Supporting Information Available

Morphology of pectin/pectin and sucralfate in different gastrointestinal pH; deteriorated PSH when adjusted to pH 11; viscosity of PSH formulated with sucralfate digested in various HCl solutions (0.1–0.5 N); frequency sweep measurements of PSH; change of rheological properties of pectin; frequency sweep measurements of pectin; SEM images of PSH; EDS analysis result for PSH and pectin; WST-1 cell viability assay for PSH and pectin; live/dead cytotoxicity assay for pectin and PSH; IVIS imaging of gastrointestinal tract from mouse gavaged with PBS; representative pictures of mice fed with different diets; 3D CT imaging of mice from various camera angles; histological analysis of the adipose tissue section; and histological analysis of the brain section (PDF)
Representative 3D CT imaging of mice from the control, HFD, and HFD + PSH groups (ZIP)

## Notes

No unexpected or unusually high safety hazards were encountered.
