# Supplementary material for: Design Strategy for a Hydroxide-Triggered pH-Responsive Hydrogel as a Mucoadhesive Barrier to Prevent Metabolism Disorders
Source: ACS Appl Mater Interfaces. 2021 Dec 6;13(49):58340–51. doi: 10.1021/acsami.1c17706 (PMC8802295; doi:10.1021/acsami.1c17706)
Supplement: Supplementary file 1 — am1c17706_si_001.pdf [file am1c17706_si_001.pdf]

Supporting Information

Design strategy for a hydroxide-triggered pH-responsive hydrogel as a mucoadhesive barrier to prevent metabolism disorders

*Rui-Chian Tang, Tzu-Chien Chen, Feng-Huei Lin**

Rui-Chian Tang
Department of Biochemical Science and Technology, College of Life Science, National Taiwan University, No. 1, Sec. 4, Roosevelt Rd., Taipei, 10617, Taiwan (ROC)

Tzu-Chien Chen, Prof. Feng-Huei Lin
Department of Biomedical Engineering, College of Medicine and College of Engineering, National Taiwan University, No. 49, Fanglan Rd., Taipei, 10672, Taiwan (ROC)
*E-mail: double@ntu.edu.tw

Prof. Feng-Huei Lin
Institute of Biomedical Engineering and Nanomedicine, National Health Research Institutes, No. 35, Keyan Rd., Zhunan, Miaoli County, 35053, Taiwan (ROC)

Supplementary Experimental Details

SEM and Energy-dispersive X-ray spectroscopy (EDS) characterization

1% w/v of Pectin and PSH were adjusted to pH 7 and lyophilized respectively. The lyophilized materials were loaded on a carbon-tape coated specimen holder. They were further subjected to platinum electroplating for better conductivity. Then, the materials were visualized using SEM (JSM-6510, JEOL) under a voltage of 10kV. EDS was further performed to determine the relative percentage of each element in the sample. For each field of view, 5 individual points were analyzed and quantified using INCAEnergy software (Oxford Instruments).

Titration

Each material was titrated by adding a 1N NaOH solution gradually from an acidic environment. No more than 20 μL of NaOH solution was added for each mL of material for fear of affecting its physiochemical properties. The pH value at each data point was measured

using a pH meter (pH6175, Jenco). Each data point was plotted with added alkalinity (mmol L^{-1}) as the x-axis.

Swelling property of PSH

1 mL of a 1% w/v PSH (pH 3.5) was applied uniformly to a mucin-coated membrane and tilted to a vertical position for 1 min to remove the excess material. The PSH-coated membrane was soaked in a Petri dish filled with simulated duodenal fluid (SDF, pH 3.5, containing ~ 0.616 g/L NaOH and ~6.8 g/L KH_2PO_4) and placed in a 37°C cell incubator for 30, 60, and 120 min without shaking. The wet weights of the resulting membrane were measured and plotted with time as the x-axis to assess the swelling of PSH. The wet weights before incubation were used as baselines (100%).

Water Soluble Tetrazolium Salt-1 assay (WST-1 assay)

WST-1 assay was performed to assess the effect of materials on cell viability. Following the guidelines of ISO10993, the L-929 fibroblast cell line (ThermoFisher) was selected and cultured in the Minimum Essential Medium (MEM, ThermoFisher) with 1% of Antibiotic-Antimycotic (ThermoFisher) and 10% of Fetal Bovine Serum (FBS, Gibco) in DDW. First, the L-929 cell line was seeded in 96 wells (Labserv) at a density of 10,000 cells per well. Meanwhile, materials were immersed in MEM at a concentration of 0.2 g per mL. The original MEM represented the control; ZDEC (Sigma-Aldrich) represented the positive control; Aluminum oxide (Sigma-Aldrich) represented the negative control. After 24 hours in the cell incubator, the MEM was replaced with the material solution in the 96 well. After another day of incubation with the materials, the solution was replaced with MEM containing 10% of WST-1 (Takara), and the cells were incubated for 2 hours. Eventually, the absorbance at 450 nm was measured with an ELISA reader. Results were normalized to the control (100%).

Live/dead assay

A Live/Dead assay was performed to estimate the cytotoxicity of the materials. The L-929 cell line was cultured in an identical medium during the WST-1 assay. First, the cells were seeded in 12 wells (Labserv) at a density of 20,000 cells per well. At the same time, the material solution was prepared as in the WST-1 assay. An exception was made for the positive control, where 5 µl of Triton™ X-100 was added 5 min before the medium was refreshed with the working solution to prevent the floating of the cells. After 24 hours in the cell incubator, the MEM in the 12 wells was replaced with the material solution. After another day of incubation, the material solution was replaced with the working solution (MEM with 2 µM of calcein-AM and 4 µM of EthD-1) and the cells were incubated for a further 40 min. After that, the working solution was replaced by PBS and the cells were observed with a fluorescence microscope to evaluate the cytotoxicity of PSH.

3D CT imaging

The body fat deposition of mice was visualized using high-resolution 3D X-ray microscopy based on micro-computed tomography (SKYSCAN). The spatial resolution was 35 µm voxel spacing. 3D images was rendered using CTvox software (Bruker).

Blood and histological analysis of the mice

Blood was collected from the heart immediately after sacrifice, and plasma was obtained by centrifuging the blood samples at 6000 rpm, carefully extracted, and stored at -20°C in the refrigerator. Clinical parameters such as total cholesterol (TC), triglycerides (TG), LDL, HDL, AST, ALT, and blood urea nitrogen (BUN) were analyzed using a Cobas c111 analyzer (Roche). Adipose tissue and liver were harvested immediately after sacrifice and soaked in

formalin for one week, processed for histological examination, and stained with hematoxylin and eosin stain.

Statistical Analysis

Data are presented as the arithmetic means plus or minus standard error of the mean. The data were analyzed by GraphPad Prism 8. The statistical significance of the bar plots with only one categorical variable was determined using one-way ANOVA followed by Tukey's multiple comparisons. The time-course experiments and bar plots with two independent variables were analyzed using two-way ANOVA followed by Tukey's multiple comparisons. An associated probability (p value) of less than 0.05 was considered significant and given one star, while the p value < 0.002 was given two stars, p value < 0.0001 was given 3 stars.

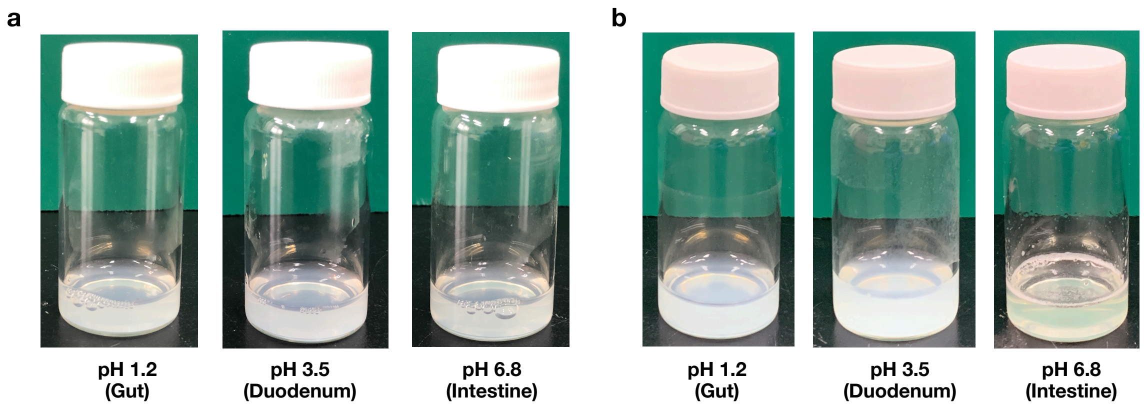

Figure S1. (a) 1% w/v pectin in different gastrointestinal pH (gut, pH 1.2; duodenum, pH 3.5; intestine, pH 6.8). (b) Solution containing 1% w/v pectin and 0.1% w/v sucralfate (without acid treatment) in different gastrointestinal pH (gut, pH 1.2; duodenum, pH 3.5; intestine, pH 6.8).

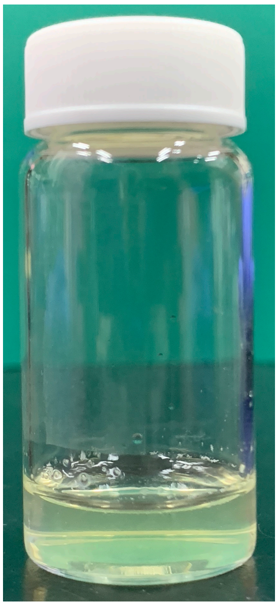

Figure S2. The deteriorated PSH when adjusted to pH 11. PSH exhibited a yellow shade and lost its pH-dependent property.

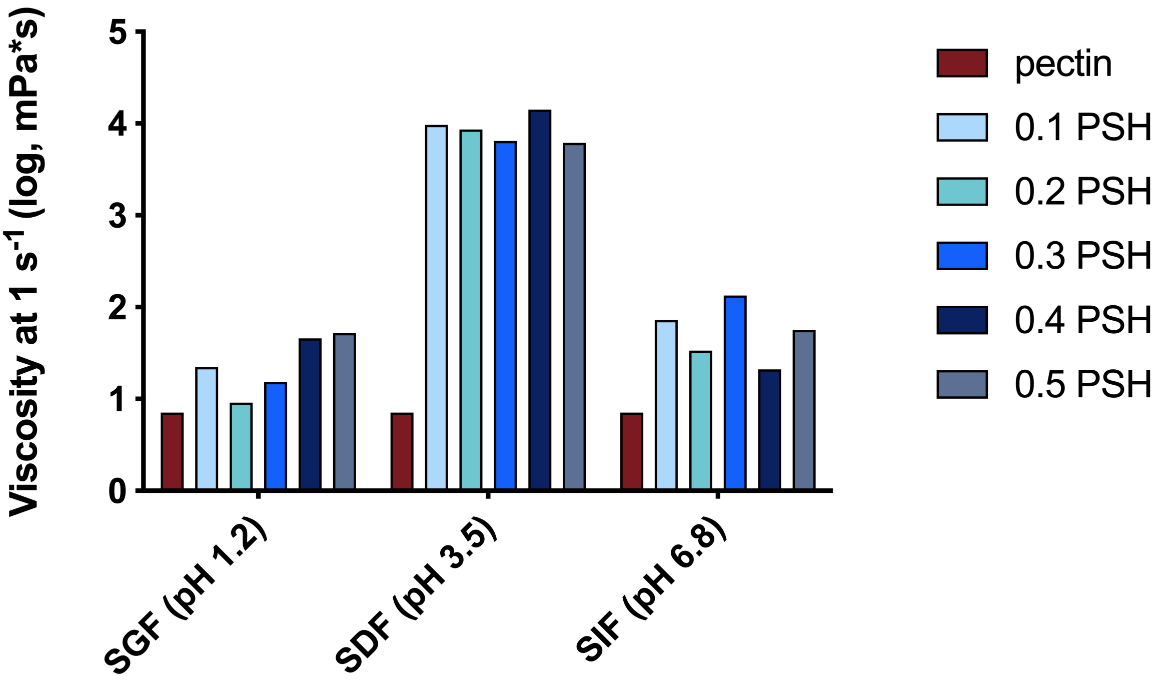

Figure S3. The viscosity of PSH formulated with sucralfate digested in various HCl solutions (0.1-0.5N) in different gastrointestinal pH (gut, pH 1.2; duodenum, pH 3.5; intestine, pH 6.8).

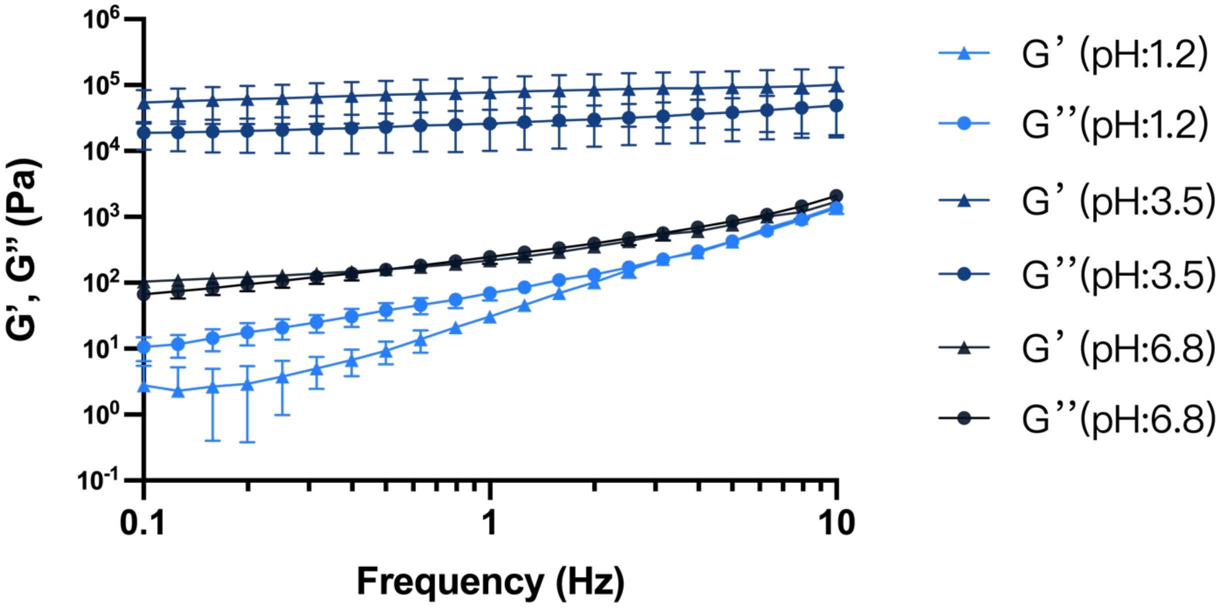

Figure S4. Frequency sweep measurements of PSH in different gastrointestinal pH (gut, pH 1.2; duodenum, pH 3.5; intestine, pH 6.8) (n = 3).

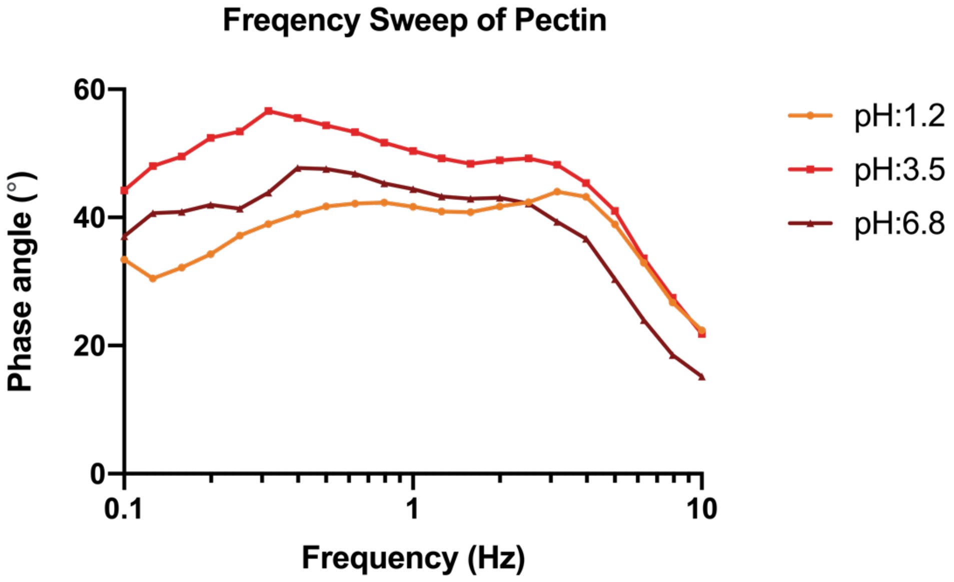

Figure S5. Change of rheological properties of pectin in different gastrointestinal pH (gut, pH 1.2; duodenum, pH 3.5; intestine, pH 6.8).

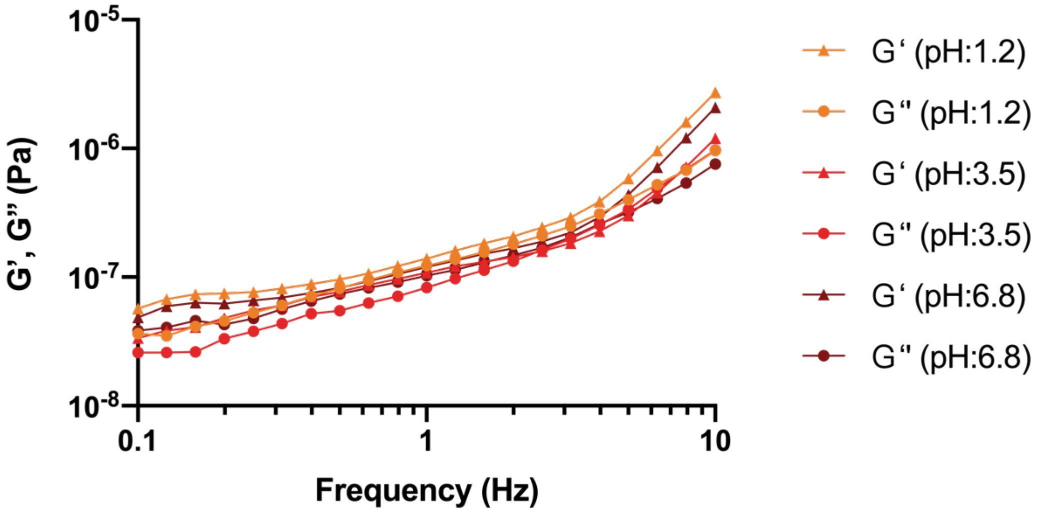

Figure S6. Frequency sweep measurements of pectin in different gastrointestinal pH (gut, pH 1.2; duodenum, pH 3.5; intestine, pH 6.8).

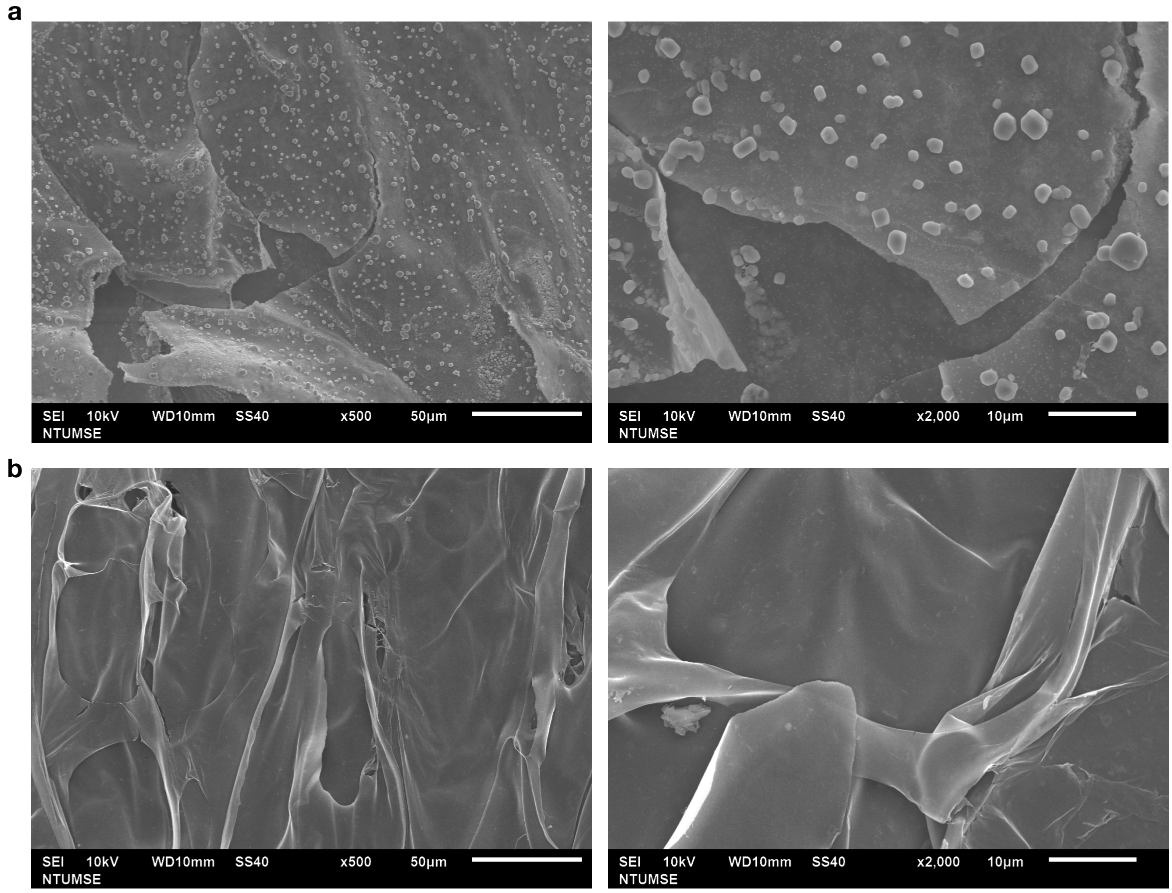

Figure S7. (a) SEM image of PSH at 500X magnification and 2000X magnification. (b) SEM image of pectin at 500X magnification and 2000X magnification.

a

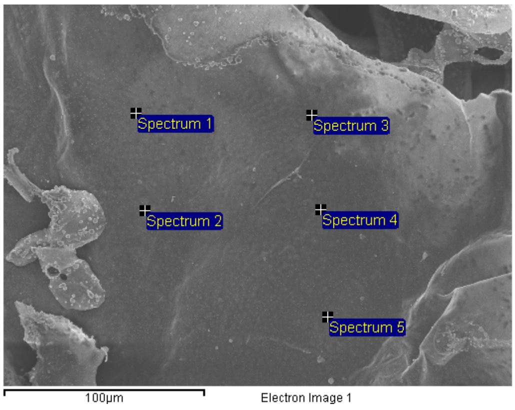

Spectrum	In stats.	C	O	Al	S
Spectrum 1	Yes	45.78	48.84	2.60	2.78
Spectrum 2	Yes	41.29	52.60	3.09	3.02
Spectrum 3	Yes	48.54	44.02	3.72	3.71
Spectrum 4	Yes	51.40	43.78	2.50	2.32
Spectrum 5	Yes	53.47	41.91	1.38	3.24
Mean		48.10	46.23	2.66	3.01
Std. deviation		4.79	4.39	0.86	0.52
Max.		53.47	52.60	3.72	3.71
Min.		41.29	41.91	1.38	2.32

b

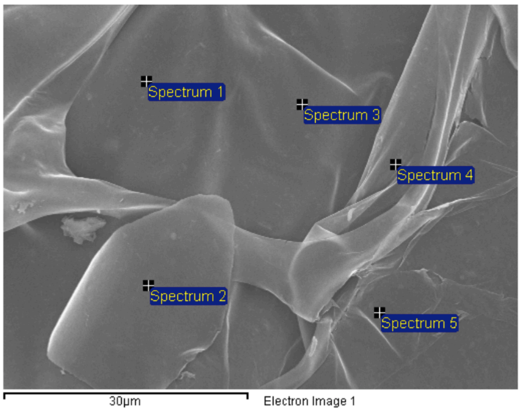

Spectrum	In stats.	C	O	Al	S
Spectrum 1	Yes	47.46	52.28	-0.09	0.34
Spectrum 2	Yes	49.49	50.34	-0.01	0.18
Spectrum 3	Yes	48.26	51.51	-0.14	0.37
Spectrum 4	Yes	47.75	51.67	0.23	0.35
Spectrum 5	Yes	49.20	50.53	-0.05	0.32
Mean		48.44	51.26	-0.01	0.31
Std. deviation		0.89	0.82	0.14	0.07
Max.		49.49	52.28	0.23	0.37
Min.		47.46	50.34	-0.14	0.18

Figure S8. (a) EDS analysis result for PSH. (b) EDS analysis result for pectin.

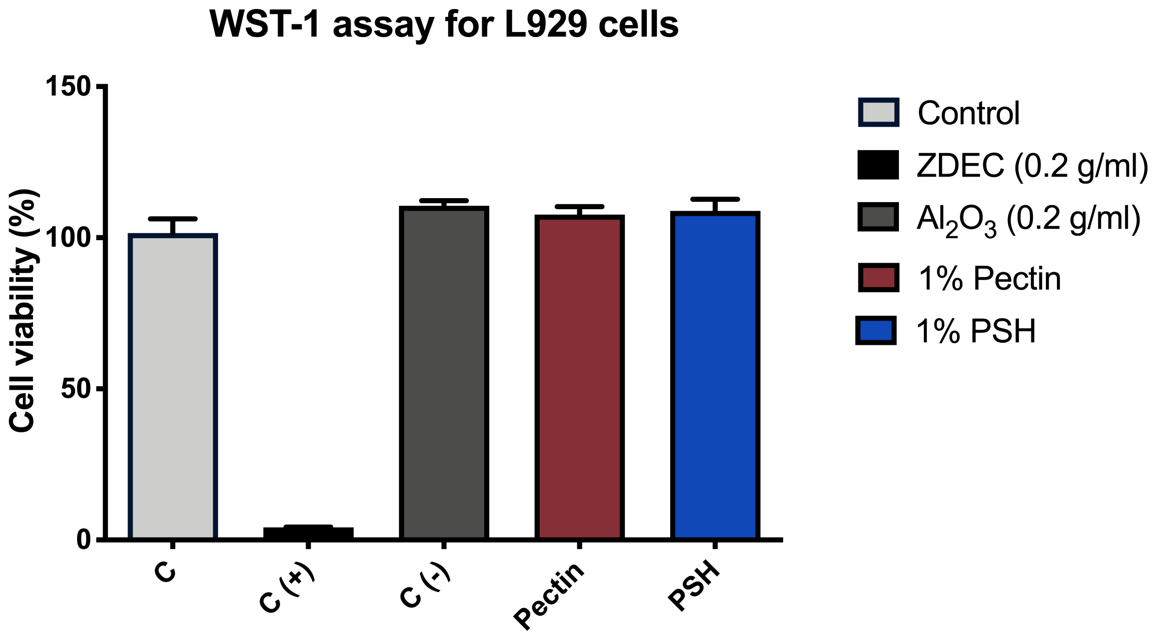

Figure S9. WST-1 cell viability assay for PSH and pectin based on ISO-10993 standards (*n* = 6). The absorbance at 450 nm was measured and the results were normalized to the control.

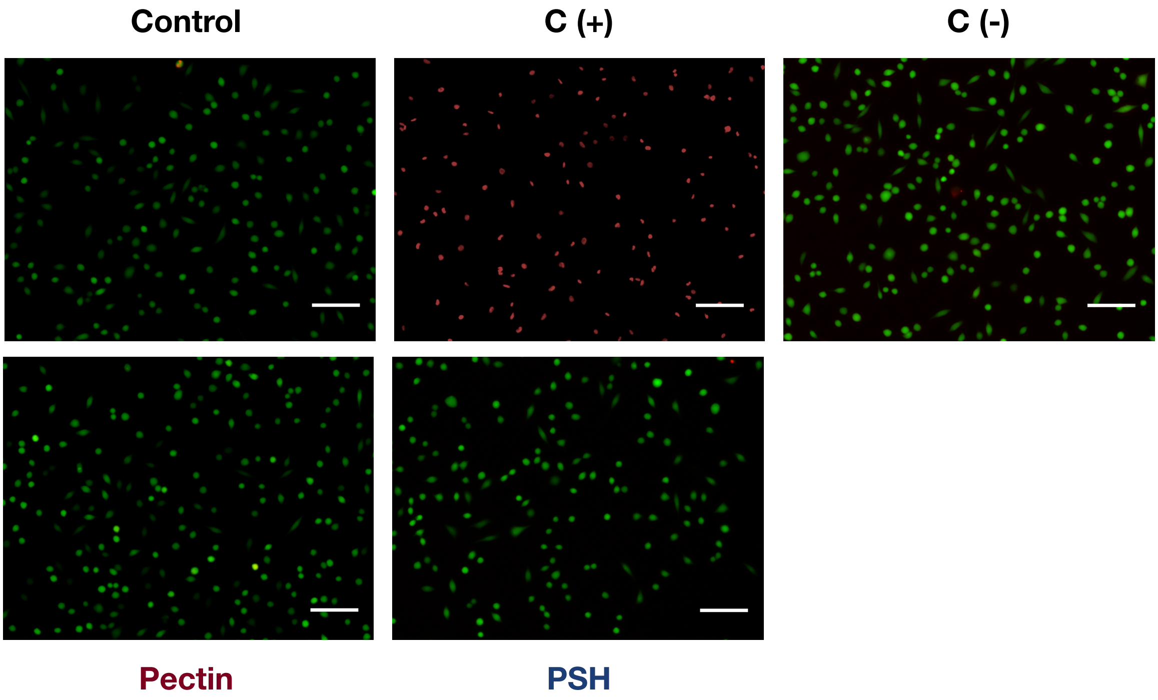

Figure S10. Live/dead cytotoxicity assay for pectin and PSH. The living cells were stained with calcein-AM (green); the dead cells were stained with EthD-1 (red); the nucleus was stained with Hoechst (blue). Scale bar, 100 μm.

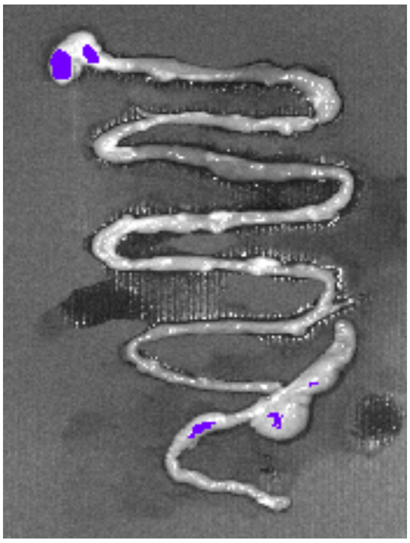

Figure S11. IVIS imaging of gastrointestinal tract from mouse gavaged with PBS only (control group).

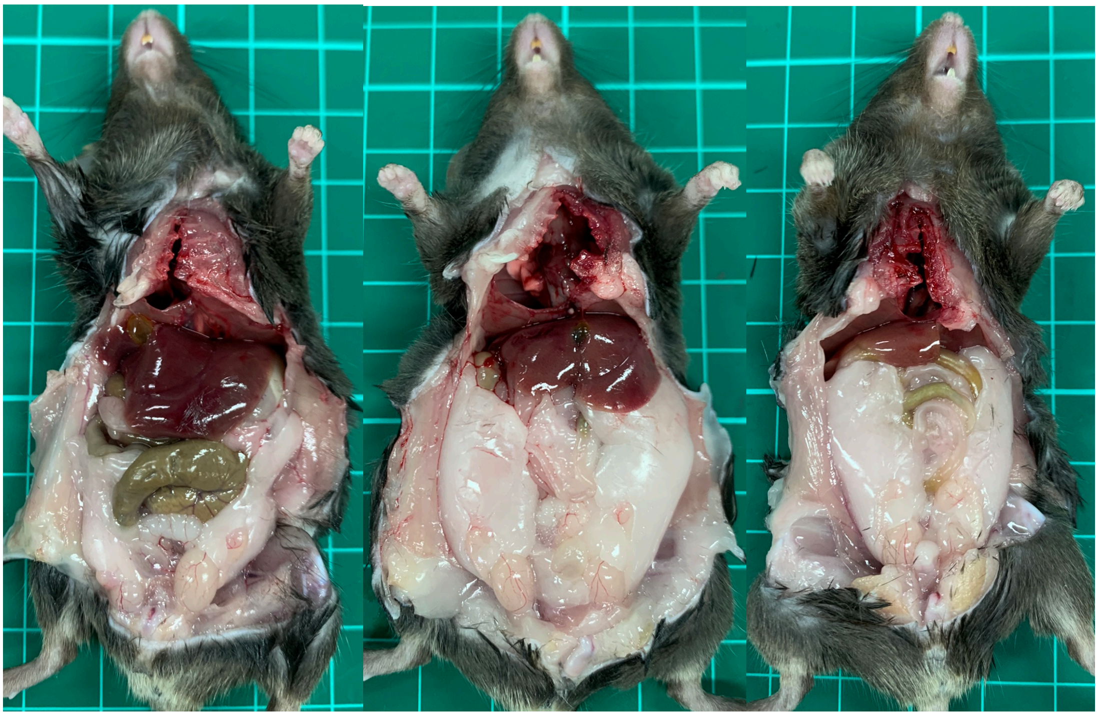

Figure S12. Representative pictures of mice fed with rodent chow (control), HFD, and HFD+PSH.

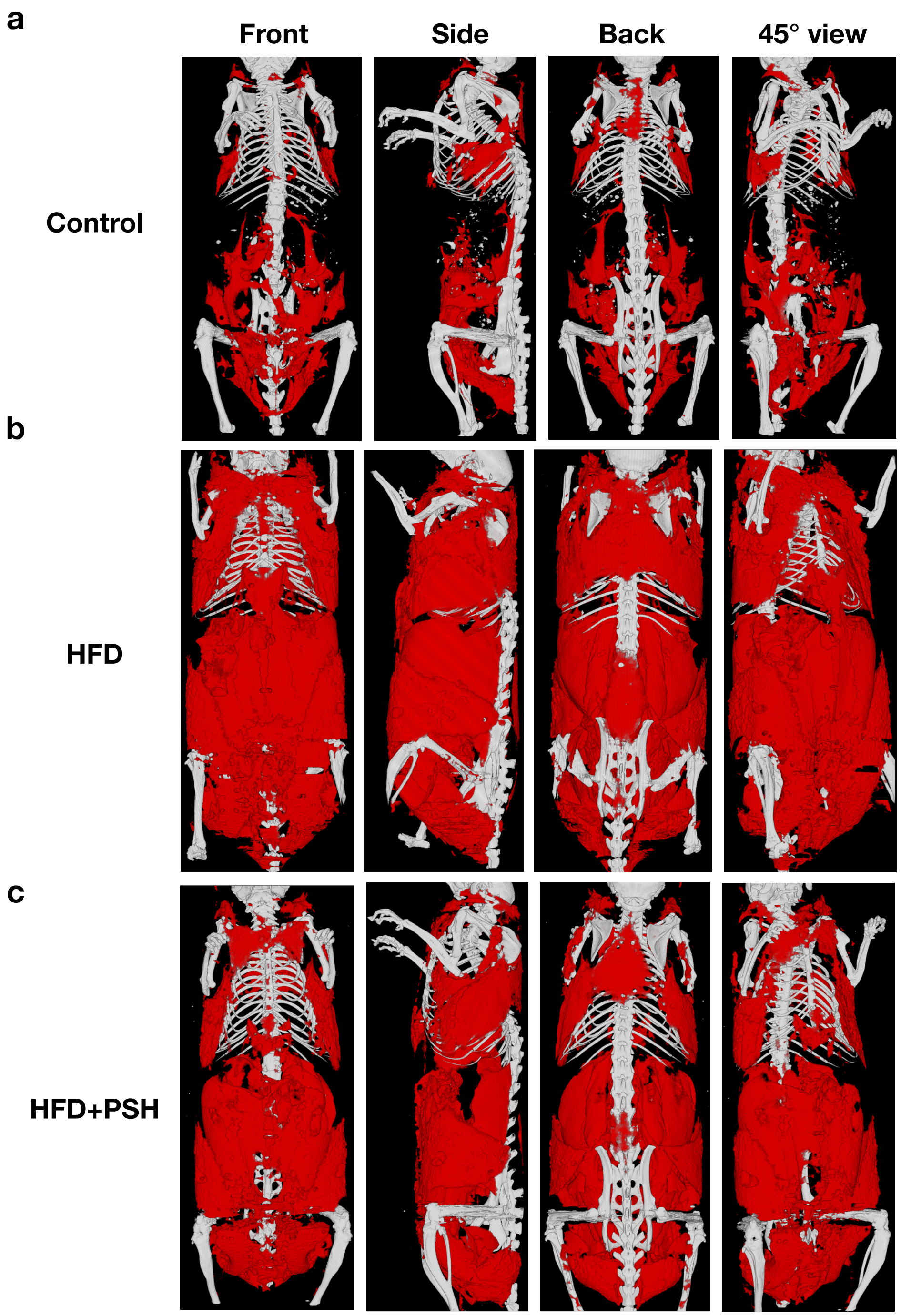

Figure S13. 3D CT imaging of mice from various camera angles with a resolution of 35 µm voxel spacing. Skeletons were represented in white; adipose tissue was represented in red. (a) Representative image of the control. (b) Representative image of the HFD. (c) Representative image of the HFD+PSH.

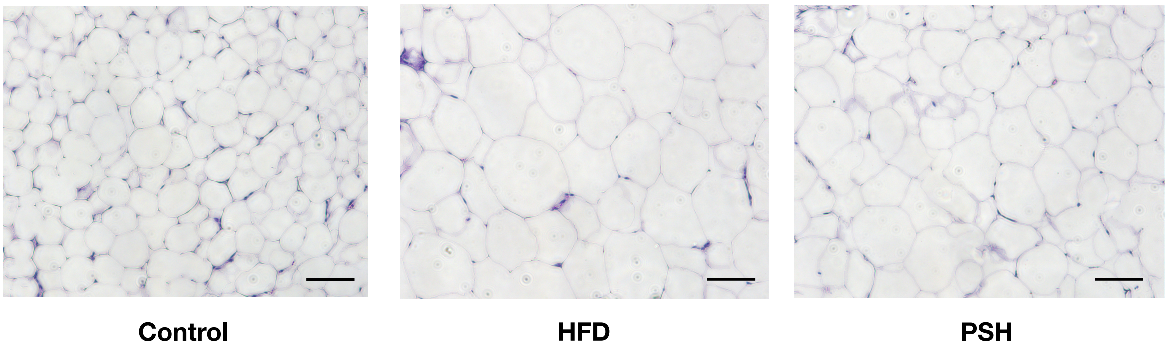

Figure S14. Histological analysis of adipose tissue section in eWAT. Scale bar, 100 μm.

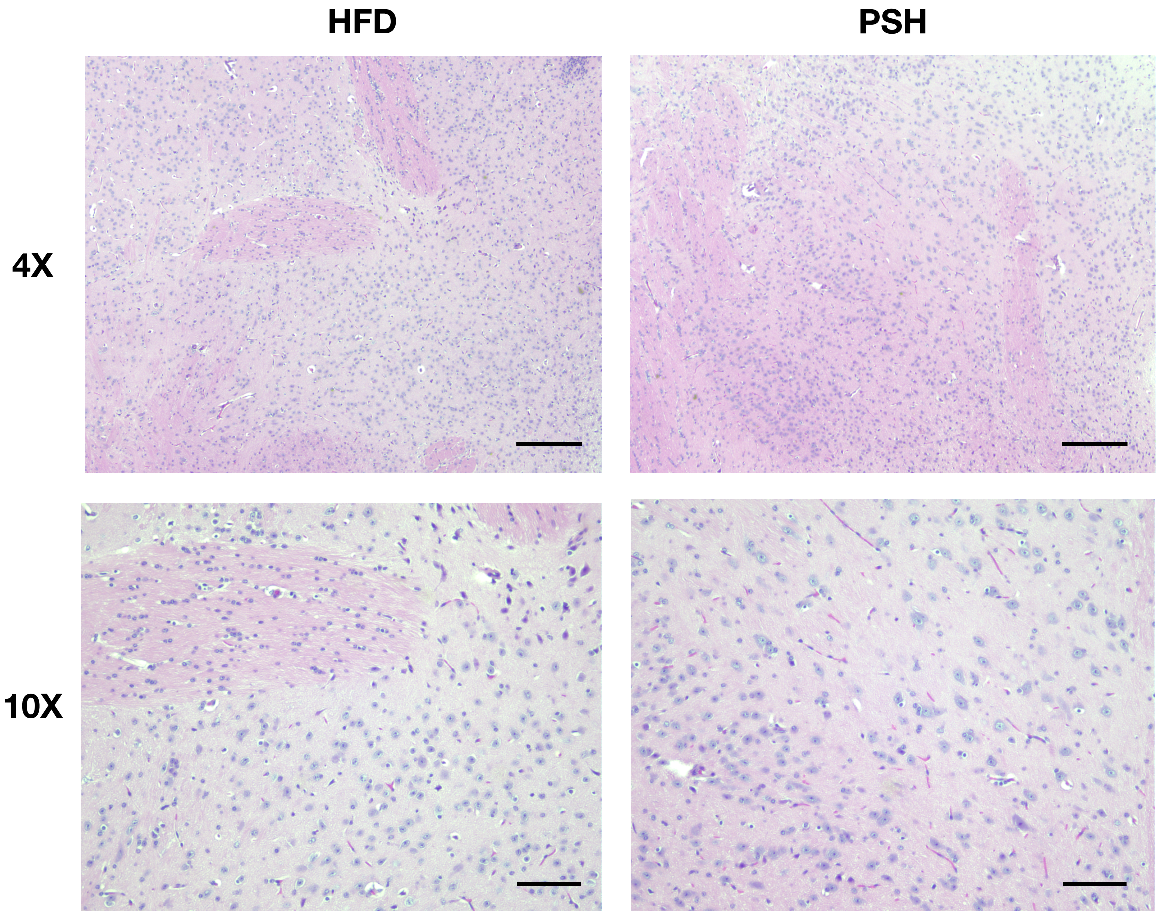

Figure S15. Histological analysis of brain section. Scale bar, 250 μm for 4X view; 100 μm for 10X view.

Movie S1. Representative 3D CT imaging of a mouse from the control group.

Movie S2. Representative 3D CT imaging of a mouse from the HFD group.

Movie S3. Representative 3D CT imaging of a mouse from the HFD+PSH group.
